# Role of interleukin-1 and tumour necrosis factor in leukocyte recruitment to acute dermal inflammation

**DOI:** 10.1155/S0962935192000528

**Published:** 1992

**Authors:** Andrew C. Issekutz, Nancy Lopes, Thomas B. Issekutz

**Affiliations:** Departments of Pediatrics Microbiology and Immunology Dalhousie University 5850 University Avenue, Nova Scotia Halifax B3J 3G9 Canada

## Abstract

The cytokines IL-1 and TNF-α are involved in inflammation and their production is stimulated by various agents, especially endotoxin (LPS). Here, using the human IL-1 receptor antagonist (IL-1RA) and a new monoclonal antibody (mAb 7F11) to rabbit TNF, the role of endogenous IL-l and TNF production in acute (3h) leukocyte (PMNL) recruitment to dermal inflammation in rabbits has been studied. IL-1RA inhibited by 27% the PMNL accumulation in reactions induced by killed Escherichia coli (p < 0.05) but not by LPS. The monoclonal antibody to TNF inhibited by 27% and 38% (p < 0.002) the PMNL accumulation in LPS and E. coli reactions respectively, but a combination of the mAb with IL-1RA was not more effective. Treatment of human umbilical vein endothelium with LPS for 3 h activated endothelium to induce PMNL transendothelial migration in vitro, which was not inhibited by IL-1RA, antibody to TNF-α, IL-1 or to IL-8. In conclusion, TNF and IL-1 may partially mediate acute PMNL infiltration in vivo to LPS and Gram negative bacteria, but there is a major IL-1/TNF independent mechanism, at least in dermal inflammation, which may be due to direct LPS activation of the microvasculature or perhaps the generation of cytokines other than IL-1 and TNF.


Mediators of Inflammation, 1, 347–353 (1992)

### Role of interleukin-1 and tumour necrosis factor in leukocyte recruitment to acute dermal inflammation

Andrew C. Issekutz,[CA] Nancy Lopes and Thomas B. Issekutz

Departments of Pediatrics, Microbiology and Immunology, Dalhousie University, 5850 University Avenue, Halifax, Nova Scotia, Canada B3J 3G9

[CA] Corresponding Author

**THE cytokines IL-1 and TNF-α are involved in inflammation and their production is stimulated by various agents, especially endotoxin (LPS). Here, using the human IL-1 receptor antagonist (IL-1RA) and a new monoclonal antibody (mAb 7F11) to rabbit TNF, the role of endogenous IL-1 and TNF production in acute (3 h) leukocyte (PMNL) recruitment to dermal inflammation in rabbits has been studied. IL-1RA inhibited by 27% the PMNL accumulation in reactions induced by killed *Escherichia coli* ($p < 0.05$) but not by LPS. The monoclonal antibody to TNF inhibited by 27% and 38% ($p < 0.002$) the PMNL accumulation in LPS and *E. coli* reactions respectively, but a combination of the mAb with IL-1RA was not more effective. Treatment of human umbilical vein endothelium with LPS for 3 h activated endothelium to induce PMNL transendothelial migration *in vitro*, which was not inhibited by IL-1RA, antibody to TNF-α, IL-1 or to IL-8. In conclusion, TNF and IL-1 may partially mediate acute PMNL infiltration *in vivo* to LPS and Gram negative bacteria, but there is a major IL-1/TNF independent mechanism, at least in dermal inflammation, which may be due to direct LPS activation of the microvasculature or perhaps the generation of cytokines other than IL-1 and TNF.**

**Key words:** IL-1, Inflammation, Interleukin, Neutrophil, TNF

### Introduction

The acute inflammatory reaction is usually characterized by rapid, early (1–2 h) infiltration of the tissue by polymorphonuclear leukocytes (PMNL) and an increase in vascular permeability. Subsequently, vascular and tissue injury may result.[1] A host of mediators and mediator systems have been implicated in this response, including vasoactive amines, lipids and proteins, some of which are generated by activation of cascade systems such as the complement and coagulation pathways.[2] More recently, some members of the cytokine family of proteins have been found to have inflammatory properties.[3–6] Among these, interleukin-1 (IL-1) and tumour necrosis factor α (TNF) are synthesized rapidly, especially by monocytes, resident tissue macrophages and to a lesser extent by PMNL, lymphocytes, and connective tissue cells.[7,8] Both IL-1 and TNF can subsequently active other cells, such as vascular endothelium to become adhesive for PMNL and mononuclear leukocytes, and to induce the synthesis of cytokines directly chemotactic for leukocytes such as IL-8 and monocyte chemotactic protein-1 (MCP-1).[6,9] Among the cytokines, IL-1 and TNF-α, are likely the earliest cytokines in the cytokine cascade to be synthesized during acute inflammatory reactions.

It is now well recognized that vascular and tissue injury during acute inflammation is in part dependent on the invasion of the involved tissues by PMNL.[1] As part of on-going investigations into the mechanisms regulating PMNL migration into inflammation, an acute dermal inflammation model in rabbits has been used in this study. The role of endogenously produced IL-1 and TNF in the acute PMNL accumulation in dermal inflammatory reactions induced by a variety of agents has been investigated. For this purpose, recombinant human IL-1 receptor antagonist (IL-1RA)[10,11] and a new monoclonal antibody (mAb) which neutralizes rabbit TNF, were employed to block the activity of endogenously produced rabbit IL-1 and TNF. The results indicate that of the reactions studied, TNF in particular, contributes significantly but only to a minor extent, to the PMNL infiltration during killed *Escherichia coli* and endotoxin (LPS) induced inflammation.

### Materials and Methods

*Dermal inflammatory reactions:* New Zealand White rabbits of either sex (3–4 kg) had their backs shaved the day before experiments. On the day of study, $^{51}Cr$ labelled blood leukocytes (see below), were injected i.v. and up to 56 skin sites were injected intradermally (i.d.) (0.2 ml) with inflammatory stimuli in a random pattern using 30-gauge needles as described previously.[12,13] Three hours later, the

© 1992 Rapid Communications of Oxford Ltd animal was sacrificed with an intravenous (i.v.) overdose of pentobarbital, the skin was removed, and the lesions punched out using a 16 mm leather punch. Radioactivity in the skin samples was determined in an LKB gamma spectrometer.

*Inflammatory stimuli:* Recombinant human interleukin-1 (IL-1α), which had a specific activity of $4 \times 10^7$ U/mg, was a gift from Dr D. Urdal (Immunex Corp., Seattle, WA, USA). Recombinant human tumour necrosis factor (TNF-α) (specific activity of $5 \times 10^7$ U/mg) was a gift from Genentech Inc. (South San Francisco, CA, USA). All of these cytokines contained $\leq 1$ ng of endotoxin/mg. Each of the cytokines were diluted immediately before use in 0.1% endotoxin-free human serum albumin (Connaught Labs., Don Mills, Ontario, Canada) in phosphate buffered saline (HSA–PBS). *E. coli* 0111 lipopolysaccharide (LPS) was from List Biologicals (Campbell, CA). *E. coli* 018.K1H7, an isolate from an infant with meningitis, was grown in broth culture, killed with formaldehyde, washed, and the number of bacteria were estimated spectrophotometrically as described previously.[12] Zymosan activated plasma (ZAP) was generated as previously[14] by incubation of heparinized (5 U/ml) rabbit plasma for 60 min (37°C) with 5 mg/ml Zymosan A (Sigma Chemical Co., St. Louis, MO, USA) followed by removal of zymosan by centrifugation.

The reversed dermal Arthus reaction was induced by i.v. injection of 10 mg human pyrogen free IgG (Sandoglobulin, Sandoz Pharmaceuticals, Dorval, Quebec, Canada), 1 h prior to i.d. injection of heat inactivated hyperimmune rabbit antiserum to human IgG.[13,15] Rabbit TNF-α was purified from rabbit macrophage culture supernatants. Briefly, rabbit peritoneal macrophages were harvested, following pentobarbital euthanasia, by peritoneal lavage with 300 ml Tyrodes solution (2 U/ml heparin), 5 days following i.p. injection of 35 ml sterile light mineral oil.[16] The cells were adhered in 175 $cm^2$ tissue culture flasks (Falcon, Fisher Scientific Co., Dartmouth, Nova Scotia, Canada) for 2 h in RPMI-1640 (Gibco, Grand Island, NY, USA) 5% heat inactivated rabbit serum, then rinsed to remove non-adherent cells. The adherent cells, which were >95% macrophages, were stimulated with LPS (30 ng/ml) in RPMI-1640 for 6 h at 37°C in 5% $CO_2$. The supernatant was then harvested, dialysed against water, concentrated by lyophilization 100–200-fold and applied to a Superose-12 FPLC column (Pharmacia Fine Chemicals, Dorval, Quebec) equilibrated with 0.3 M NaCl–0.01 M $Na_2HPO_4$, pH 7. Fractions eluting between 40 and 50 kilodaltons (kDa), were rich in TNF activity as assayed by cytotoxicity for the TNF sensitive L929 mouse fibroblast line.[17] The fractions which eluted between 12 and 20 kDa were devoid of IL-1 activity.[18]

*Anticytokine reagents:* Highly purified human recombinant interleukin-1 receptor antagonist (IL-1RA) was a kind gift from Dr R. C. Thompson (Synergen Inc., Boulder, CO). The rat mAb 7F11, which neutralized rabbit macrophage TNF-α and lymphocyte derived TNF-β bioactivity, was rat isotype $IgG_1$ according to a commercially available isotyping kit (Serotec Canada, Toronto, Ontario). This antibody was generated by immunization of AO strain male rats with partially purified TNF-α (40–50 kDa Superose-12 fractions). The immunization protocol consisted of covalently coupling 3–5 μg of protein to 1 $cm^2$ pieces of the APT form of Transabind paper (Schleicher and Schuell Ltd, Keene, NH, USA) according to the instructions of the manufacturer. The immobilized antigen was introduced surgically under anaesthesia into the peritoneal cavity of the rats every 10–14 days together with i.p. injection of polyadenosine–polyuridine (800 μg) (Sigma Chemical Co.) as an adjuvant.[19,20] After a total of four such immunizations, the animals had neutralizing activity in their serum for rabbit TNF-α. After a rest period, one rat was booster immunized by intrasplenic injection of 5 μg of partially purified antigen. Four days later the spleen was removed, and splenocytes were fused with the P3U1 mouse myeloma using polyethylene glycol 1450 (Merck Frosst, Montreal, Quebec) according to standard techniques.[21] Following fusion, the lymphocytes were plated into flat bottom 96-well microtitre plates (NUNC, Gibco) at a density of $2 \times 10^5$ cells/well in Dulbecco Modified Eagle's Medium (DMEM; Gibco) with non-essential amino acids, Na pyruvate and 10% foetal calf serum (FCS; Hyclone, Logan, UT, USA), into wells containing a mixture of $5 \times 10^5$ irradiated mouse thymocytes and splenocytes prepared 24 h previously as feeder cells.[21] Supernatants from hybridoma-containing wells were screened for neutralization of the cytotoxic activity of rabbit TNF-α, and one of the positive hybridomas, 7F11 was cloned by limiting dilution. This mAb neutralized both rabbit macrophage derived TNF-α and mitogen (Con A and PHA) stimulated rabbit lymphocyte derived TNF activity, presumably TNF-β.[7] However, mAb 7F11 was specific for rabbit TNF in that it did not neutralize mouse, rat or human TNFα activity. The mAb was purified by affinity chromatography on Protein G Sepharose from cells grown in serum free medium (DMEM supplemented with 2% Nutridoma, Boehringer Mannheim, Montreal, Quebec). Binding of the IgG was at pH7 in PBS and elution was with 0.1 M glycine–HCl, pH 3.5 followed by pH 2.7 buffers, as recommended by the manufacturer (Pharmacia Fine

Chemicals, Dorval, Quebec). The purified mAb preparation had a titre of 30 000 neutralizing units/ml.

Polyclonal rabbit antibody to human IL-1$\alpha$ and IL-1$\beta$ and to human TNF-$\alpha$ were kind gifts from Dr C. Dinarello (Tufts University, Boston, MA, USA). Polyclonal goat antibody to human IL-8 was a gift from Sandoz Pharmaceuticals (Vienna, Austria). These antibodies were all neutralizing and were used in a dilution of 1:100 in the *in vitro* assay system. Neutralizing mAb to human TNF-$\alpha$ was a kind gift from Dr M. Sheppard (Genentech Inc., South San Francisco, CA). It had a neutralizing titre of $8 \times 10^6$ NU/mg.

*Measurement of rabbit leukocyte accumulation in dermal inflammation:* The quantitation of leukocyte accumulation in dermal inflammatory reactions has been described in detail previously.[13,18] Briefly, 30 ml of acid–citrate dextrose anticoagulated blood was collected from the rabbit and red blood cells were removed by hydroxyethyl cellulose sedimentation (Fluka Chemical Co., Ronkonkoma, NY). The leukocytes were labelled with $Na_2{}^{51}CrO_4$ (Amersham Inc., Oakville, Ontario), washed with $Ca^{2+}$, $Mg^{2+}$ free Tyrodes' buffer–10% ACD plasma, and injected i.v. just prior to the injection (i.d.) of inflammatory stimuli in skin sites. It has been shown[13,18] that results with this mixed leukocyte population, labelled with $^{51}Cr$ are comparable to that obtained with purified $^{51}Cr$-labelled PMNLs, as long as the leukocyte infiltration into the acute inflammatory lesions is composed of $>90\%$ PMNLs and no local haemorrhage in the site occurs. Both of these criteria were met in this work during the period of study of the lesions (3 h). The $^{51}Cr$ cpm in the skin lesions was converted to number of PMNLs $\times 10^6$ accumulated/site by standardizing values relative to the response to zymosan activated plasma (ZAP) at $10 \times 10^6$ PMNL/site. This value is based on the mean response ($10 \pm 0.8 \times 10^6$ PMNL/site; SEM) in 30 rabbits, in previous studies in which blood PMNL specific activity (number of PMNL/cpm) was determined after $^{51}Cr$–leukocyte injection, using hydroxyethylcellulose sedimentation of red cells and Percoll's density gradient purification of the PMNLs. The PMNL specific activity measurement allowed for conversion of $^{51}Cr$ content in skin sites to number of PMNL/site as described.[13,18]

*Endothelial cell cultures:* Human umbilical vein endothelial cells (HUVE) were obtained using collagenase digestion as described by Jaffe *et al.*[22] and described in detail previously.[23] Briefly, isolated cells were grown to confluence in gelatin coated flasks in RPMI-1640-20% FCS, supplemented with Endothelial Cell Growth Supplement (25 $\mu$g/ml) (Collaborative Research, Lexington, MA), Na pyruvate (1 mM), 2-mercaptoethanol (2ME) (50 $\mu$M), heparin (90 $\mu$g/ml) and penicillin/streptomycin. HUVE were then gently trypsinized and plated on gelatin–fibronectin coated polycarbonate filters bearing 3 $\mu$m pores in 24-well transwell culture plate inserts (6.5 mm) (Costar Corp., Cambridge, MA). After 6 days of culture in the above medium, endothelium formed a tight monolayer and permeability barrier separating an upper chamber of 100 $\mu$l volume from a lower chamber (24-well plate macrowell) containing 600 $\mu$l medium as described previously.[23]

*Human polymorphonuclear leukocyte purification:* Human PMNL were purified from acid citrate dextrose–heparin anticoagulated peripheral venous blood of healthy donors as described previously.[23] Briefly, red cells were sedimented with 6% dextran–saline (Abbott Labs, Montreal, Quebec), leukocyte rich plasma was collected and leukocytes were labelled with $Na_2{}^{51}CrO_4$ (Amersham Inc.). PMNLs were then purified by discontinuous Percoll's gradient centrifugation, washed, and resuspended to $3 \times 10^6$ PMNL/ml in RPMI-1640-0.5% HSA-10mM Hepes pH 7.2. This method yielded PMNL of $\geq 95\%$ purity with essentially no red cell contamination and $\geq 98\%$ cell viability.

In vitro *PMNL transendothelial migration assay:* For assays, HUVE monolayers were handled according to the previously published protocol.[23] In this study, HUVE were stimulated by addition to the lower chamber of various concentrations of LPS in fresh RPMI-1640-10% FCS. After stimulation, monolayers and upper and lower chambers were rinsed, the medium in the lower chamber was exchanged (600 $\mu$l) for RPMI-1640-HSA-Hepes, and the $^{51}Cr$-PMNL suspension (100 $\mu$l) was loaded above the monolayer/filter, and incubated at 37°C in 5% $CO_2$. After 75 min, migration was stopped by washing the surface above the monolayer with RPMI-1640 to remove non-adherent PMNLs, and the undersurface of the filter was rinsed with 2 ml of ice-cold PBS–0.2% EDTA solution and collected into the lower compartment. The PMNLs which migrated into the lower compartment and those which detached from the undersurface of the filter by washing were lysed with 0.5% Triton X 100 to release the $^{51}Cr$. The $^{51}Cr$ content of the lower compartment (medium + PBS–EDTA wash of filter undersurface) represented migrated PMNLs. The results are expressed as a percentage of total added $^{51}Cr$-PMNL in the lower compartment.

*Statistical analysis:* Statistical analysis of results was performed by 'paired' Student's *t*-test.

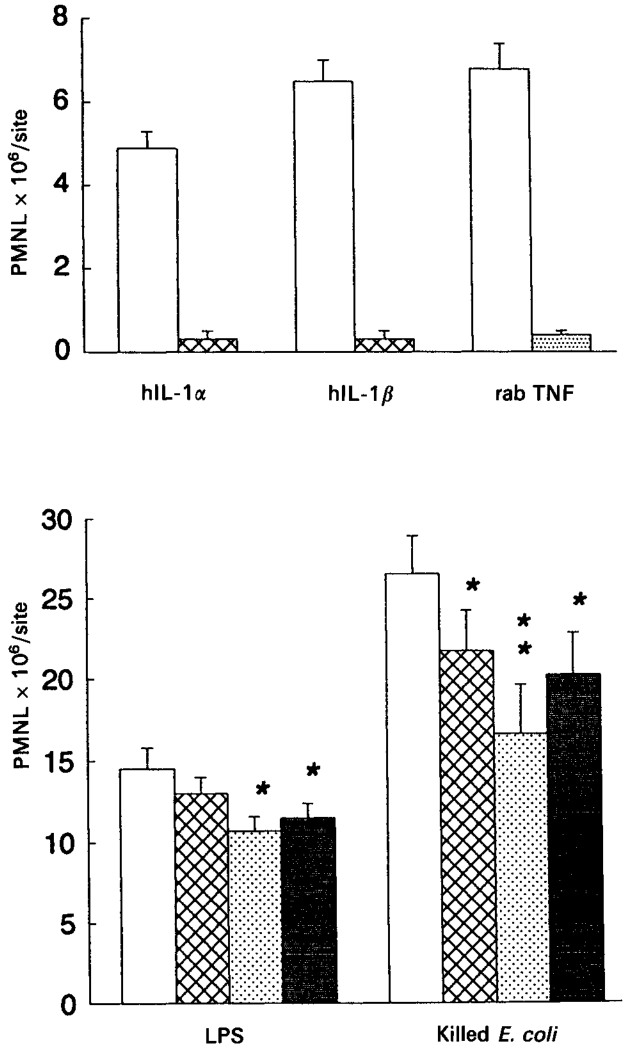

FIG. 1. The effect of IL-1 receptor antagonist and antibody to TNF on dermal PMNL accumulation. Skin sites on rabbits were injected (i.d.) with human IL-1α, IL-1β (2 ng/site), rabbit TNF-α (500 U/site), *E. coli* LPS (5 ng/site) or killed *E. coli* ($10^7$/site) together with control (HSA–PBS or irrelevant mAb), IL-1RA (400 ng), mAb to TNF (1000 NU), or both reagents in parallel in each rabbit. PMNL accumulation was quantitated with $^{51}$Cr-PMNL for 3 h after initiation of the reactions. Values are means ±SEM of five experiments performed with triplicate or quadruplicate replicates. *$p < 0.05$; **$p < 0.002$ by paired Student's *t*-test. □, control; ☒, IL-1RA; ▒, mAb to TNF; ■, IL-1RA+mAb to TNF.

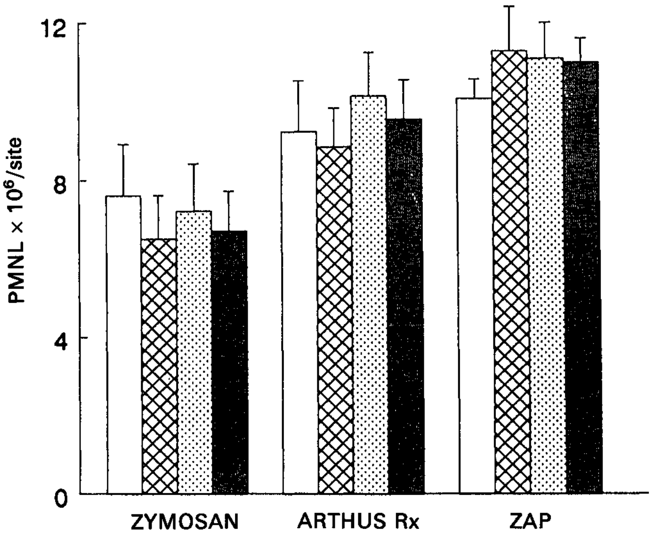

FIG. 2. The effect of IL-1 receptor antagonist and antibody to TNF on dermal PMNL accumulation. Skin sites were injected i.d. with zymosan A (2 mg), zymosan activated plasma (ZAP, 25%) or antiserum to human IgG (1:8) following i.v. injection of human IgG, to induce the reversed Arthus reaction. Other sites were injected with these agents in combination with IL-1RA or mAb to TNF as in Fig 1. Values are means ±SEM of three experiments. Key as for Fig 1.

### Results

*Effect of IL-1 receptor antagonist and antibody to TNF on dermal PMNL infiltration:* In initial experiments the efficacy of recombinant human IL-1 receptor antagonist (IL-1RA) and the mAb to rabbit TNF for blocking the *in vivo* activity of IL-1, and rabbit TNF-α for inducing PMNL accumulation in skin sites were tested. As can be seen in Fig. 1, the injection of 2 ng of recombinant human IL-1α or IL-1β or partially purified rabbit macrophage derived TNF-α, induced marked PMNL accumulation during the first 3 h following i.d. injection. Control injections of HSA–PBS (data not shown) did not cause PMNL accumulation (0.2 ± 0.05 × $10^6$; uninjected skin 0.15 ± 0.02 × $10^6$ PMNL per site). These HSA–PBS control values were subtracted from all the values shown in Figs 1 and 2. The simultaneous injection of IL-1RA in a dose of 400 ng per site, together with either IL-1α or IL-1β, almost completely blocked the PMNL accumulation in these sites. Similarly, the addition of mAb to rabbit TNF-α at a dose of 1000 NU per injection site, virtually eliminated the PMNL recruiting activity of 500 units of rabbit TNF-α. In cross inhibition studies the rabbit mAb to TNF did not inhibit the activity of IL-1α or IL-1β for recruiting PMNL into skin sites and vice versa, the human IL-1RA did not inhibit (<10% inhibition) the activity of rabbit TNF-α for inducing PMNL accumulation (not shown).

We next investigated the effects of IL-1RA and mAb to TNF on the accumulation of PMNL in skin sites in the rabbits injected with *E. coli* LPS or killed *E. coli* bacteria. As can be seen on the lower panel in Fig. 1, the leukocyte accumulation induced by LPS was not significantly inhibited by the co-injection of IL-1RA, but was significantly inhibited by mAb to rabbit TNF. The combination of IL-1RA with mAb to TNF did not result in greater inhibition than did mAb to TNF alone. In response to i.d. injection of killed *E. coli*, both IL-1RA and TNF mAb significantly inhibited PMNL accumulation. The combination of IL-1RA with anti-TNF did not inhibit to any greater extent than either alone. In these experiments PMNL accumulation was measured during the first 3 h after i.d. injection of the skin sites, since it has been shown previously that maximum PMNL accumulation occurs during this time in these types of lesions.[12] All combinations of agents were studied in the same rabbit with triplicate or quadruplicate skin sites being injected and statistical analysis was by paired *t*-test. The dose of IL-1RA and mAb to TNF were optimized for inhibitory activity and the level of inhibition with IL-1RA did not increase upon increasing the dose of IL-1RA to 1000 ng/site or by increasing the mAb dose to 2500 NU per site.

The effect of IL-1RA and mAb to TNF on three other inflammatory reactions was also studied, namely that induced by i.d. injection of zymosan particles, by the reversed passive Arthus reaction and by ZAP, the active principle of which is $C5a_{des\ Arg}$.[14] As shown in Fig. 2, neither IL-1RA nor TNF mAb, nor a combination of both agents inhibited the PMNL accumulation induced by zymosan, ZAP or the Arthus reaction.

*The effect of LPS on PMNL migration across vascular endothelium:* The migration of PMNL into inflammatory reactions involving IL-1 or TNF, are believed to involve the IL-1 and TNF activation of vascular endothelium to express adhesion molecules for PMNL (reviewed in Ref. 9). Various authors have shown that this activated endothelium not only promotes PMNL adhesion but also PMNL transendothelial migration *in vitro*.[9,23] Gram negative bacterial LPS has also been show to activate endothelium for adhesion of PMNL, but the role of endothelium generated cytokines such as IL-1 or TNF, which could directly activate the endothelium, is not clear.[9] Therefore, the effect of LPS on PMNL interaction directly with vascular endothelial cells was studied using an *in vitro* system which allows quantitation of PMNL transendothelial migration. Endothelium stimulated with LPS for 3 h resulted in PMNL transendothelial migration as shown in Fig. 3, even though the LPS was removed from the system. An LPS concentration of 0.3 ng/ml was sufficient to induce a maximal response. These results are comparable to previous findings using this system with IL-1 or TNFα to activate the endothelium, i.e. 2–4 h of stimulation was optimal for observing PMNL transendothelial migration and periods less than 2 h or longer than 8 h resulted in a decrease in the migration response[23] (and not shown). In contrast to the 3 h required to activate endothelium with LPS for supporting this transendothelial migration, PMNL migration across the activated endothelium was rapid, plateauing between 60 to 90 min (not shown) as it was with IL-1 and TNF-α induced PMNL migration reported previously.[23] There was no migration of PMNL in response to any of the concentrations of LPS, in the absence of an endothelial monolayer[24] (and not shown).

It has been shown previously that endothelium can be activated to secrete cytokines such as IL-1, IL-8 and possibly TNF.[6,9] Therefore, the possibility was examined that the observed effects of LPS may have been due to the endogenous production of one of these cytokines by the endothelium and secondary activation of the endothelium in an autocrine fashion for supporting PMNL migration. Table 1 shows the effects of IL-1RA, and antibodies to IL-1α, IL-1β, TNF-α or IL-8 on LPS stimulated PMNL transendothelial migration. The IL-1RA and antibodies were present during the 3 h LPS stimulation and also during the 75 min PMNL migration, to ensure maximal neutralization of any endogenously produced IL-1, TNF or IL-8.

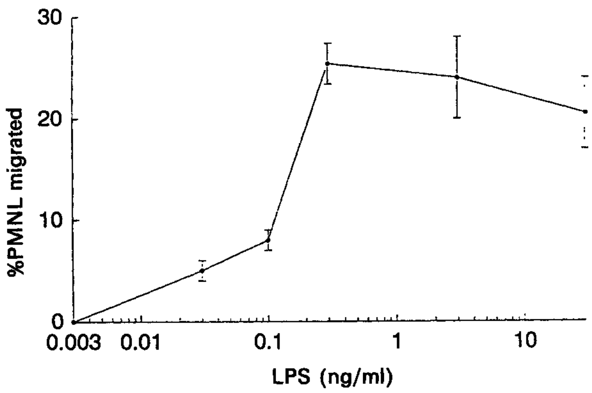

FIG. 3. The effect of LPS on human PMNL transendothelial migration. Human umbilical vein endothelial cell monolayers grown on filters were stimulated for 3 h with various concentrations of LPS, and washed. PMNL labelled with $^{51}Cr$ were added and allowed to migrate through the monolayer for 75 min. Quantitation of PMNL transendothelial migration is as in Methods. PMNL migration across unstimulated endothelium was <3% and was subtracted from each value. Values are means ±SEM of three dose–response experiments performed with triplicate replicates.

**Table 1.** The effect of blocking IL-1, TNF-α and IL-8 on LPS activation of endothelium for PMNL transendothelial migration

| Blocking agent[a] | % Inhibition of PMNL migration |
|---|---|
| IL-1RA (300 ng/ml) | 8.0 |
| mAb TNFα (500 NU/ml) | 7.5 |
| IL-1RA + mAb TNFα | 4.1 |
| anti-IL-8 IgG | 0 |
| IL-1RA + mAb TNFα + anti IL-8 | 0 |
| polyclonal antiserum to IL-1α, IL-1β, + TNFα | 7.1 |

[a] Endothelial monolayers as in Fig. 1 were stimulated with LPS (1 ng/ml) for 3 h in the presence of blocking agents or non-immune serum. After the medium was exchanged prior to the PMNL migration stage, the agents were added back and were present throughout the assay. Polyclonal antisera were used at 1:100 dilution, the predetermined dilution shown to completely block the activities of exogenously added IL-1α, IL-1β, TNFα and IL-8 in separate PMNL transendothelial migration assays (not shown). Similarly the mAb to TNFα and the IL-1RA concentrations completely blocked activation of endothelium by TNFα (10 U/ml) or IL-1α (0.3 ng/ml). Values are means of two experiments for each condition.

Although the antibodies to TNF-α, IL-1, IL-8 and IL-1RA each effectively blocked (>95%) PMNL migration induced by the corresponding exogenously applied cytokine (not shown), the LPS induced activation of endothelium for PMNL transendothelial migration was not significantly inhibited by any of the agents even in combination.

### Discussion

The major findings in this study are that IL-1RA and mAb to TNF inhibited PMNL accumulation in inflammatory reactions induced in the dermis by killed *E. coli*, mAb to TNF inhibited the LPS reaction but, even in combination, these agents only partially inhibited PMNL accumulation in these reactions (Fig. 1). This finding was surprising because IL-1 and TNF-α induce PMNL infiltration into the skin upon intradermal injection and they have synergistic effects.[3,4] The results suggest that endogenous production of TNF, at least in dermal inflammation may be more important than IL-1 in LPS and perhaps also in *E. coli* inflammatory reactions, the latter being partly dependent on LPS shed from the cell wall of the bacteria.[24] Another explanation for our results, namely that IL-1RA may not be active as an antagonist of the rabbit IL-1 receptor, is unlikely because the human IL-1RA effectively inhibited the PMNL accumulation in rabbit skin induced by i.d. injection of IL-1α and IL-1β and systemic or local treatment in various species with human IL-1RA has inhibited PMNL infiltration e.g. in LPS induced alveolitis in rats and immune complex colitis in rabbits.[10,25,26] The relative lack of effect of IL-1RA in our experiments, in comparison to these other models is unlikely to be due to the doses employed because concentrations of up to 1000 ng per site had no greater inhibitory effect than 200 ng (not shown). Since our mAb neutralizes both α and β species of TNF, it is not possible from these results to conclude that neutralization of TNF-α was responsible for the observed inhibition. However, TNF-β is produced primarily by activated lymphocytes[7] of which there are relatively few in the skin during the acute (<3 h) phase of inflammation.[27]

The differences observed in our experiments with IL-1RA and other inflammation models in which IL-1 appears to play an important role in PMNL recruitment to LPS, for example in peritonitis[28] or alveolitis[25,29] may be dependent on the tissue and the type of resident cells, especially macrophages available to secrete IL-1, a cytokine which is not readily secreted by all types of macrophages and is released slower or later than TNF-α.[8,30,31] Among skin cells, keratinocytes are known to store IL-1 but apparently do not release this except perhaps upon cellular injury.[32] Langerhans cells have been shown to secrete IL-1 and TNF-α, and mast cells have recently been shown to secrete preformed stores of TNF-α.[33,34] Secretion of TNF-α in skin from these cells may occur more rapidly than of IL-1, perhaps accounting for the greater apparent contribution of TNF to PMNL infiltration in the initial 3 h of the reaction. This conclusion is also supported by the observations that TNF-α is secreted more rapidly (<1 h) than IL-1 in response to LPS injection systemically[35] or LPS in the cerebrospinal fluid.[36]

Additional findings in the dermal inflammation model reported here suggest that neither endogenous production of IL-1 nor TNF contribute to the PMNL infiltration induced by *in situ* immune complex formation, as in the reversed passive Arthus vasculitis reaction or by zymosan particles (Fig. 2). This is not surprising in that both these reactions are primarily dependent on complement activation[15,37] and likely require the generation of $C5a_{des\,Arg}$ to induce PMNL accumulation. The PMNL recruiting activities of ZAP, which is known to be due to $C5a_{des\,Arg}$,[14] was not inhibited by IL-1RA or by mAb to TNF, as expected and this stimulus served primarily as a negative control and an internal standard.

Finally, the results in Fig. 1 suggest that for LPS and *E. coli* dermal inflammatory reactions endogenous IL-1 and TNF play minor roles in PMNL recruitment and that additional mechanisms are operative. The killed *E. coli* reaction is complex and involves, in part, complement activation by the cell wall of the bacterium with the generation of $C5a_{des\,Arg}$ chemotactic activity as well as the release of LPS from the cell wall of the bacterium, which has no direct PMNL chemotactic properties.[24] The response to LPS is also complex. However, two mechanisms can largely be excluded: (a) the LPS used here and at the nanogram concentrations which induce intense inflammatory reactions, has been shown previously not to activate the complement pathway or to generate chemotactic activity for PMNL;[24] and (b) the LPS was not directly chemotactic and did not induce PMNL migration *in vitro* in the abscence of an endothelium.[24] Endotoxin (LPS) of course is known to be a potent inducer of IL-1 and TNF-α production[4,6–8,35] and via the action of these cytokines could recruit PMNL. In addition, LPS, IL-1 and TNFα all can induce endothelium to produce the PMNL chemotactic cytokine, IL-8, which is also produced by connective tissue cells such as fibroblasts in response to IL-1 or TNF-α (but not LPS).[6,8] However, the present findings demonstrate that LPS also can directly activate endothelial cells to promote PMNL transendothelial migration (Fig. 3). This action of LPS, at concentrations ≤1 ng/ml, does not appear to involve IL-1, TNF-α or IL-8 production by the endothelial cells (Table 1). Such a direct effect of LPS on the microvasculature could, in part explain why only partial inhibition *in vivo* of PMNL infiltration by the TNF mAb and IL-1RA was observed in these studies and in other LPS induced inflammation models such as in pulmonary inflammation.[25] However, additional cytokines may also be involved in the LPS response such as the 45 kDa protein, previously reported to be produced by LPS stimulated rabbit and human macrophages, which is also active in recruiting PMNL into the tissues.[18] These conclusions of alternate IL-1 and TNF-α independent LPS induced mechanisms for PMNL recruitment are supported by most recent observations with LPS induced inflammation in monkey skin,[38] demonstrating that *in vivo* the endothelium expresses the leukocyte adhesion molecule–E selectin and PMNL infiltration occurs prior to immunohistochemical evidence of IL-1 and TNF-α production.

### References

1. Movat HZ. Chemotaxis — inflammation induced by immune complexes: a model for complement-chemotaxis-and PMN-leukocyte-medicated process. In: *The Inflammatory Reaction.* Amsterdam: Elsevier, 1985; 235–244.
2. Movat HZ. Mediators of the vascular phenomena of acute inflammation. In: *The Inflammatory Reaction.* Amsterdam: Elsevier, 1985; 77–123.
3. Wankowicz Z, Megyeri P, Issekutz A. Synergy between tumour necrosis factor α and interleukin-1 in the induction of polymorphonuclear leukocyte migration during inflammation. *J Leukoc Biol* 1988; **43**: 349–356.
4. Movat HZ, Cybulsky MI, Colditz IG, Chan MKW, Dinarello CA. Acute inflammation in gram-negative infection: endotoxin, interleukin 1, tumor necrosis factor, and neutrophils. *FASEB J* 1987; **46**: 97–103.
5. Granstein RD, Margolis R, Mizel SB, Sauder DN. *In vivo* inflammatory activity of epidermal cell-derived thymocyte activating factor and recombinant interleukin 1 in the mouse. *J Clin Invest* 1986; **77**: 1020.
6. Larsen C, Zachariae C, Mukaida N, *et al.* Proinflammatory cytokines interleukin 1 and tumor necrosis factor induce cytokines that are chemotactic for neutropils, T cells and monocytes. *Prog Clin Biol Res* 1990; **349**: 419–431.
7. Vassalli P. The pathophysiology of tumor necrosis factors. *Ann Rev Immunol* 1992; **10**: 411–452.
8. Dinarello CA. The biology of interleukin-1. *Chem Immunol* 1992; **51**: 1–32.
9. Pober JS, Cotran RS. Cytokines and endothelial cell biology. *Physiol Rev* 1990; **70**: 427–452.
10. Dinarello CA, Thompson RC. Blocking IL-1: interleukin 1 receptor antagonist *in vivo* and *in vitro. Immunol Today* 1991; **12**: 404–410.
11. Hannum CH, Wilcox CJ, Arend WP, *et al.* Interleukin-1 receptor antagonist activity of a human interleukin-1 inhibitor. *Nature* 1990; **343**: 336–340.
12. Issekutz A, Bhimji S, Bortolussi R. Effect of immune serum or polymyxin B on *Escherichia coli*-induced inflammation and vascular injury. *Infect Immun* 1982; **36**: 548–557.
13. Issekutz AC, Issekutz TB. Quantitation of blood cell accumulation and vascular responses in inflammatory reactions. In: Chang J, Lewis AJ, eds. *Modern Methods in Pharmacology.* New York: Alan R. Liss Inc., 1989; 129–150.
14. Issekutz AC, Movat HZ, Movat KW. Enhanced vascular permeability and haemorrhage-inducing activity of rabbit $C5a_{des\ arg}$: probable role of polymorphonuclear leucocyte lysosomes. *Clin Exp Immunol* 1980; **41**: 512–520.
15. Movat HZ. Chemotaxis—quantitation and kinetics of the Arthus reaction. In: *The Inflammatory Reaction.* Amsterdam: Elsevier, 1985; 245–258.
16. Mathison JC, Wolfson E, Ulevitch RJ. Participation of tumor necrosis factor in the mediation of gram negative bacterial lipopolysaccharide-induced injury in rabbits. *J Clin Invest* 1988; **81**: 1925–1937.
17. Aggarwal BB, Moffat B, Lee SH, Harkins RN. Chemical and biological properties of human lymphotoxin. In: Goldstein AL, ed. *Thymic Hormones and Lymphokines.* New York: Plenum Publishing Corporation, 1984; 235–245.
18. Issekutz AC, Megyeri P, Issekutz TB. Role for macrophage products in endotoxin-induced polymorphonuclear leukocyte accumulation during inflammation. *Lab Invest* 1987; **56**: 49–59.
19. Hovanessian AG, Galabru J, Rivière Y, Montagnier L. Efficiency of poly(A).poly(U) as an adjuvant. *Immunol Today* 1988; **9**: 161–162.
20. Viamontes GI, Audhya T, Goldstein G. Antibodies to thymopoietin following implantation of paper disks derivatized with synthetic Cys-thymopoietin[28–39]. *J Immunol Methods* 1986; **94**: 13–17.
21. Harlow E, Lane D. Monoclonal antibodies—producing hybridomas. In: *Antibodies, A Laboratory Manual.* Cold Spring Harbor Laboratory, 1988; 196–228.
22. Jaffe EA, Nachman RL, Becker CG, Minick CR. Culture of human endothelian cells derived from umbilical veins. Identification by morphological and immunologic criteria. *J Clin Invest* 1973; **52**: 2745–2752.
23. Morzycki W, Sadowska J, Issekutz AC. Interleukin-1 and tumour necrosis factor α induced polymorphonuclear leukocyte–endothelial cell adhesion and transendothelial migration *in vitro*: the effect of apical versus basal monolayer stimulation. *Immunol Lett* 1990; **25**: 331–340.
24. Issekutz AC, Bhimji S. Role for endotoxin in the leukocyte infiltration accompanying *Escherichia coli* inflammation. *Infect Immun* 1982; **36**: 558–566.
25. Ulich TR, Yin S, Guo K, del Castillo J, Eisenberg SP, Thompson RC. The intratracheal administration of endotoxin and cytokines. III. The interleukin-1 (IL-1) receptor antagonist inhibits endotoxin and IL-1 induced acute inflammation. *Am J Pathol* 1991; **138**: 521–524.
26. Cominelli F, Nast CC, Clark BD, *et al.* Interleukin 1 (IL-1) gene expression, synthesis, and effect of specific IL-1 receptor blockade in rabbit immune complex colitis. *J Clin Invest* 1990; **86**: 972–980.
27. Issekutz AC, Movat HZ. The *in vivo* quantitation and kinetics of rabbit neutrophil leukocyte accumulation in the skin in response to chemotactic agents and *E. coli. Lab Invest* 1980; **42**: 310–318.
28. McIntyre KW, Stepan GJ, Kolinsky KD, *et al.* Inhibition of interleukin 1 (IL-1) binding and bioactivity *in vitro* and modulation of acute inflammation *in vivo* by IL-1 receptor antagonist and anti-IL-1 receptor monoclonal antibody. *J Exp Med* 1991; **173**: 931–939.
29. Warren JS. Intrapulmonary interleukin 1 mediates acute immune complex alveolitis in the rat. *Biochem Biophys Res Commun* 1991; **175**: 604–610.
30. Issekutz AC, Morzycki W, Sadowska J. Rabbit alveolar macrophages stimulated with endotoxin and lung fragments from endotoxemic rabbits produce a leukocyte infiltration-inducing factor that lacks IL-1, TNFα, or chemotactic activity. *Exp Lung Res* 1991; **17**: 803–819.
31. Lonnemann G, Endres S, Van der Meer JWM, Cannon JG, Koch KM, Dinarello CA. Differences in the synthesis and kinetics or release of interleukin 1α, interleukin 1β and tumor necrosis factor from human mononuclear cells. *Eur J Immunol* 1989; **19**: 1531–1536.
32. McKenzie RC, Sauder DN. The role of keratinocyte cytokines in inflammation and immunity. *J Invest Dermatol* 1990; **95**: 105S–107S.
33. Larrick JW, Morhenn V, Chiang YL, Shi T. Activated langerhans cells release tumor necrosis factor. *J Leukoc Biol* 1989; **45**: 429–433.
34. Gordon JR, Galli SJ. Mast cells as a source of both preformed and immunologically inducible TNF-Alpha/cachectin. *Nature* 1990; **346**: 274–276.
35. Chensue SW, Terbuh PD, Remick DG, Scales WE, Kunkel SL. *In vivo* biologic and immunohistochemical analysis of interleukin-1 alpha, beta and tumor necrosis factor during experimental endotoxemia. *Am J Pathol* 1991; **138**: 395–402.
36. Waage A, Galstensen A, Shalaby R, Brandtzaeg P, Kierulf P, Espevik T. Local production of tumor necrosis factor α, interleukin 1, and interleukin 6 in meningococcal meningitis. *J Exp Med* 1989; **170**: 1859–1867.
37. Movat HZ, Cybulsky MI. Neutrophil emigration and microvascular injury. Role of chemotaxins, endotoxin, interleukin-1 and tumour necrosis factor α. *Surv Synth Pathol Res* 1987; **6**: 153–176.
38. Ringler DJ, Walsh DG, MacKey JJ, McEver RP, Hunt RD, Newman W. Sequential cutaneous cytokine localization, leukocyte-endothelial cell adhesion molecule (CAM) expression, and leukocyte typing in rhesus monkeys receiving intradermal LPS. *FASEB J* 1992; **6**: A1889.

ACKNOWLEDGEMENTS. The authors are grateful for gifts of cytokines from Dr D. Urdal (Immunex Corp.), Dr M. Sheppard (Genentech Inc.), Dr R. C. Thompson (Synergen Inc.), Dr C. Dinarello (Tuft's University), and from Sandoz Pharmaceuticals, which helped make this study possible. We are also grateful for the excellent technical assistance of Ms L. Dunlop and Mr K. MacLeod, Ms J. Stoltz, and Ms D. Randell for manuscript preparation.

This work was supported by grants MA-7684 and MA-8575 from the Medical Research Council of Canada.

**Received 27 July 1992; accepted 8 August 1992**
